# Acute phase reactants in the follow-up of patients with FMF

**DOI:** 10.1186/1546-0096-13-S1-P115

**Published:** 2015-09-28

**Authors:** ZS Arıcı, ED Batu, E Sönmez, Y Bilginer, R Topaloğlu, S Özen

**Affiliations:** 1 Hacettepe University, Pediatric Rheumatology, Ankara, Turkey

## Introduction

Familial Mediterranean Fever (FMF) is the most common periodic fever syndrome. FMF characterized by recurrent fever, serositis attacks and chronic subclinical inflammation in attack-free periods.

## Objective

The aim of this study was to evaluate the relevance of acute phase reactants (APR) in FMF and to determine their correlation with each other during attacks and attack-free periods.

## Methods

Twenty-three children diagnosed as FMF according to the previously published criteria and followed-up at the Pediatric Rheumatology Clinic of Hacettepe Children's Hospital were enrolled in the study. The erythrocyte sedimentation rate (ESR), C reactive protein (CRP), white blood cell (WBC) count, platelet count, and serum amyloid a (SAA) were tested in the patients during an attack and in-between attacks.

## Results

There were 9 male and 14 female patients. Tests were performed in 11 patients with an attack and in 12 without attacks. All patient had homozygous or compound heterozygous FMF-associated mutations. ESR, CRP, WBC, SAA were statistically significantly higher in patients with an attack (respectively p < 0,001, p <0,001, p=0.03, p=0,003). Highly significant and perfect correlation was found between SAA and CRP in patients with attack (r= 0,939, p<0,001).

There was a significant correlation between the number of attacks in the last 6 months and SAA in the patient with attack-free periods (r=0,746, p=0,005). There was no other significant correlation.

## Conclusion

CRP and SAA levels correlated with each other during the FMF attacks. SAA is important to identify subclinical inflammation in FMF patients when other APRs were normal.

